# Draft Genome Sequence of an Antarctic Isolate of the Black Yeast Fungus *Exophiala mesophila*

**DOI:** 10.1128/MRA.00142-19

**Published:** 2019-05-09

**Authors:** Claudia Coleine, Laura Selbmann, Sawyer Masonjones, Silvano Onofri, Laura Zucconi, Jason E. Stajich

**Affiliations:** a Department of Ecological and Biological Sciences, University of Tuscia, Viterbo, Italy; b Mycological Section, Italian National Antarctic Museum (MNA), Genoa, Italy; c Department of Microbiology and Plant Pathology, University of California—Riverside, Riverside, California, USA; Vanderbilt University

## Abstract

A 30.43-Mb draft genome sequence with 10,355 predicted protein-coding genes was produced for the ascomycete fungus Exophiala mesophila strain CCFEE 6314, a black yeast isolated from Antarctic cryptoendolithic communities. The sequence will be of importance for identifying differences among extremophiles and mesophiles and cataloguing the global population diversity of this organism.

## ANNOUNCEMENT

Black yeasts are a polyphyletic morphoecological group of fungi classified in either the Chaetothyriales order of class Eurotiomycetes or class Dothideomycetes (phylum Ascomycota; subphylum Pezizomycotina). They are distinguished by high melanin content, thick and multilayered cell walls, and an extraordinary ability to survive in extremes and tolerate chemical and physical stresses, such as extreme pH and temperatures, desiccation, UV ionizing radiation, and alpha particles (1–8).

Within the Herpotrichiellaceae family (Chaetothyriales), there are many recognized species in the genus Exophiala which are adapted to a multitude of ecological niches, including human environments (9, 10). Isolates from oligotrophic water sources, such as sinks, drainpipes, swimming pools, bathing facilities, and drinking water, have been described (11, 12). Species in this genus have been explored for their potential in bioremediation applications (13, 14), and several species have been isolated from glaciers (15) and microbial ecosystems specialized to extreme temperature and aridity, such as Antarctic endolithic communities (16–18). We assembled a draft genome sequence of an Antarctic strain to provide resources for comparative studies of adaptation and evolution of this intriguing group of fungi.

Exophiala mesophila strain CCFEE 6314 was provided by the Culture Collection of Fungi from Extreme Environments (CCFEE) of the Mycological Section of the Italian Antarctic National Museum. The culture was isolated from a cryptoendolithic community at Mt. Billing (71°15′S, 163°00′E) on continental Antarctica. The rock sample was collected using a sterile chisel and preserved at −20°C until the strain was isolated by directly plating fragments of colonized rock on petri dishes containing 2% malt extract agar (MEA). The pure culture was grown on 2% MEA medium plates for 6 weeks at 10°C and DNA extracted from the total biomass following the cetyltrimethylammonium bromide (CTAB) protocol (19). Melanin was removed through two phenol-chloroform purification steps. Genomic DNA was sheared with Covaris S220 ultrasonicator and sequencing library constructed using a NeoPrep TruSeq Nano DNA sample prep kit (Illumina) in the University of California—Riverside Genomics Core.

A total of 2.9 million 2 × 300-bp paired-end sequence reads were obtained from a multiplexed library from one Illumina MiSeq flow cell. A quality check of reads was performed with FastQC (v0.11.3) (20), followed by genome assembly with MaSuRCA (v2.3.2) (21), using default parameters (cgwErrorRate = 0.15), which included quality-based read trimming and corrections. Trimmed reads averaged 198 bp. Genome scaffolds were filtered of vector contamination with Sequin (v15.10) (https://www.ncbi.nlm.nih.gov/Sequin/) and redundant scaffolds eliminated if completely aligned with at least 95% identity to a longer contig using MUMmer (v3.23) (22) as implemented in “funannotate clean” in Funannotate (v0.5.5) (23). The assembly was 30.43 Mb in total length (number of contigs, 207; *N*_50_ value, 522 kb; maximum contig size, 1.43 Mb; *L*_50_ value, 20; GC content, 50%; coverage, 54×).

The Funannotate (v0.5.5) (23) pipeline was used to annotate the genome. Briefly, consensus gene models were produced by EVidenceModeler (EVM) (24) using *ab initio* predictions from AUGUSTUS (v3.2.2) (25) and GeneMark.hmm-ES (v4.32) (26) combined with protein-to-genome alignments from Exonerate (v2.2.0) (27). Self-training for GeneMark.hmm-ES was performed using default parameters, AUGUSTUS was trained with alignments of the BUSCO ascomycota_odb9 data set (v9) (28), and prediction parameters were archived (29). Protein annotations were assigned by similarity to Pfam (30) and CAZy domains (31, 32) using HMMER (3.1b2) (33), MEROPS (34), eggNOG (v4.5) (35), InterProScan (v5.20-59.0) (36), and Swiss-Prot (37) by BLASTP (v2.5.0+) (38) searches using Funannotate defaults. A total of 10,355 protein-coding genes were predicted and prepared for GenBank submission by the Genome Annotation Generator (39).

### Data availability.

This whole-genome shotgun project has been deposited at DDBJ/ENA/GenBank under the accession number NAJM00000000. The version described in this paper is the first version, NAJM01000000. The Illumina sequence reads were released under SRA accession number SRR5223779 and associated with BioProject number PRJNA342238.
